# Ramadan fasting reduces high-sensitivity C-reactive protein among HIV-infected patients receiving antiretroviral therapy

**DOI:** 10.3389/fnut.2022.964797

**Published:** 2023-01-06

**Authors:** Alvina Widhani, Evy Yunihastuti, Siti Setiati, Fiastuti Witjaksono, Teguh H. Karjadi

**Affiliations:** ^1^Allergy and Clinical Immunology Division, Department of Internal Medicine, Faculty of Medicine, Dr. Cipto Mangunkusumo General Hospital, Universitas Indonesia, Central Jakarta, Jakarta, Indonesia; ^2^HIV Integrated Clinic, Dr. Cipto Mangunkusumo General Hospital, Central Jakarta, Jakarta, Indonesia; ^3^Geriatric Division, Department of Internal Medicine, Faculty of Medicine, Dr. Cipto Mangunkusumo General Hospital, Universitas Indonesia, Central Jakarta, Jakarta, Indonesia; ^4^Center for Clinical Epidemiology and Evidence Based Medicine, Faculty of Medicine, Dr. Cipto Mangunkusumo General Hospital, Universitas Indonesia, Central Jakarta, Jakarta, Indonesia; ^5^Department of Clinical Nutrition, Faculty of Medicine, Dr. Cipto Mangunkusumo General Hospital, Universitas Indonesia, Central Jakarta, Jakarta, Indonesia

**Keywords:** Ramadan, fasting, C-reactive protein, inflammation, HIV

## Abstract

**Background:**

Inflammatory conditions and oxidative stress increase in HIV infection, and inflammation increases the risk of cardiovascular disease. Ramadan fasting is known to reduce inflammation and oxidative stress in diabetic patients. This study examined the effects of Ramadan fasting on high-sensitivity C-reactive protein (hs-CRP) levels and total antioxidant status (TAOS) in HIV patients on antiretroviral therapy (ART).

**Methods:**

This was a prospective cohort study comparing HIV-infected patients on stable ART who fasted throughout Ramadan to HIV-infected patients who did not fast during Ramadan. Inclusion criteria were men aged 20–40 years, taking first-line ART for at least 6 months, Muslims intent to fast for Ramadan, no current hospitalization because of acute conditions and not being treated for opportunistic infections.

**Results:**

After 2 weeks, hs-CRP had decreased significantly in the fasting group (−0.41 mg/L [IQR = −1; 0.10]) compared to the non-fasting group (0.20 mg/L [IQR = −0.30; 1.50]) (*p* = 0.004). The linear regression analysis has shown that Ramadan fasting contributed to 10.10% of the variance in hs-CRP value (*R*^2^ = 0.101) and decreased its value by 0.317 points (*B* = −0.317). Changes in TAOS did not significantly different (*p* = 0.405) between the fasting group (0.05 mmol/L [IQR = −0.03; 0.12]) and the non-fasting group (0.04 mmol/L [IQR = −0.13; 0.36]). In the fasting group, there were significant changes in polyunsaturated fatty acid consumption (*p* = 0.029), body weight (*p* = 0.001), cigarette smoking (*p* = 0.001), and sleeping duration (*p* = 0.001).

**Conclusion:**

Ramadan fasting reduces hs-CRP concentrations among HIV patients on ART.

## 1. Introduction

Human immunodeficiency virus (HIV) infection causes chronic immune activation and increases inflammatory cytokine release. Antiretroviral therapy (ART) can limit but not completely normalize these inflammatory conditions. Inflammation increases the risk of cardiovascular disease, metabolic complications, and malignancies (1). ART and HIV infection alone can also increase oxidative stress (2). Total antioxidant status (TAOS) has been shown to decline in HIV patients receiving ART (3).

Ramadan fasting, a type of intermittent fasting in which Muslims abstain from food and drink from dawn until sunset during the holy month of Ramadan, may reduce inflammation. Ramadan fasting has been shown to reduce high-sensitivity C-reactive protein (hs-CRP) levels significantly in patients with metabolic syndrome (4) and to improve oxidative stress. After fasting, malondialdehyde levels significantly decrease and glutathione levels increase in both normal people and diabetic patients (5). During the second trimester in pregnant women, TAOS increases significantly after 10 days of Ramadan fasting (6).

We hypothesized that Ramadan fasting may limit inflammation and oxidative stress among HIV patients receiving ART. To our knowledge, no studies have explored this effect of fasting, so in this study, we investigated the effects of Ramadan fasting on hs-CRP concentration and TAOS in HIV patients on ART.

## 2. Materials and methods

### 2.1. Study design and population

A prospective cohort study design was used to examine the effects of Ramadan fasting on high-sensitivity C-reactive protein (hs-CRP) levels and total antioxidant status (TAOS) in HIV patients on antiretroviral therapy (ART). The data collected from HIV patients on ART who fasted during Ramadan for 14 consecutive days were compared with a group of non-fasting HIV patients also on ART. The participants were recruited at the HIV Integrated Clinic of Dr. Cipto Mangunkusumo Hospital, Jakarta, Indonesia, and were followed up for 14 days. The inclusion criteria for the fasting group were HIV patients receiving first-line ART for at least 6 months, men aged 20–40 years, Muslims intent to fast for Ramadan, no current hospitalization because of acute conditions, and not being in the initial phase of treatment for an opportunistic infection (e.g., tuberculosis, cytomegalovirus, toxoplasmosis, or *Pneumocystis carinii* pneumonia). Patients who consumed steroids or other immunosuppressants and those with poor ART adherence (less than 95%) were excluded from the study. Those who fasted for less than the two consecutive weeks were classified as drop-outs from the study. HIV patients who were not planning to do Ramadan fasting and did not fulfill any of the exclusion criteria were recruited as participants in the non-fasting group. Participants in fasting and non-fasting groups were matched by age, ART regimen, and duration of ART treatment.

### 2.2. Study procedures

Participants were selected by consecutive sampling. Demographic data and other information (e.g., route of HIV transmission, history of cigarette smoking, hepatitis B or hepatitis C coinfection, ART combination, duration of ART treatment, antioxidant supplement intake, CD4 cell counts, and HIV RNA level) were collected at the start of the study.

Some data were collected both before and after the 2 weeks of follow-up. These data included ART adherence, food intake, physical activity, body weight, waist circumference, anxiety and depression assessment, and sleep quality assessment. Dietary assessment was performed at enrollment and after 2 weeks of follow up using a 3-day food record, 24-h food recall, and semi-quantitative food frequency questionnaire to record food intake. The dietary data were analyzed by the Indonesian food composition database and the nutrisurvey software. Anxiety and depression were evaluated using the Hospital Anxiety and Depression Scale (HADS) questionnaire. The HADS questionnaire comprises 7 items related to anxiety and 7 items related to depression, which are scored 0–3 for each item. The score range for either anxiety or depression was, therefore, 0–21. Sleep quality was assessed using the Pittsburgh Sleep Quality Index (PSQI), which is a self-report questionnaire of sleep quality within 1 month. The PSQI measures several aspects of sleep, including subjective sleep quality, sleep latency, sleep duration, habitual sleep efficiency, sleep disturbances, use of sleep medication, and daytime dysfunction. Each item has a 0–3 interval scale. The global PSQI score is calculated by the summation of 7 component scores, resulting in an overall score in the ranged of 0–21 where a higher score reflects poorer sleep quality.

High-sensitivity C-reactive protein concentration and TAOS were assessed before and after the 2-week follow-up. Blood was collected at 9.00–11.00 a.m. local time. Serum hs-CRP concentration was measured using CRP (latex) high-sensitivity immunoturbidimetric assay on a Cobas Integra instrument (Roche diagnostics, Indianapolis, IN, US). The normal range for hs-CRP is ≤10 mg/L. TAOS was measured using a colorimetric assay on an ADVIA 1800 instrument (Siemens Healthcare GmbH, Erlangen, Germany). The normal range for TAOS is 1.30–1.77 mmol/L.

### 2.3. Statistical analysis

Statistical analyses were performed using IBM SPSS statistics (version 20.0.0; IBM Corp., Armonk, NY, USA). Data were expressed as mean and standard deviation (for normally distributed data) and median (interquartile range for data with skewed distributions). If the data were distributed normally, comparisons of the results before and after fasting in each group and between the two groups were analyzed using paired or independent *t*-tests. Otherwise, the comparisons were analyzed using the non-parametric Wilcoxon rank-sum test and the Mann–Whitney *U* test, *p*-values < 0.05 were considered statistically significant. Linear regression analysis was used to determine the percentage of change of the main outcome variables (hs-CRP and TAOS).

### 2.4. Ethics statement

All participants gave written informed consent before study enrollment. Ethical approval was granted by the Research Ethics Board of the Faculty of Medicine, University of Indonesia (487/UN2.F1/ETIK/2015) and the Research Ethics Board of Dr. Cipto Mangunkusumo Hospital, Jakarta, Indonesia.

## 3. Results

### 3.1. Baseline characteristics

At the beginning of the study, the fasting group included 30 HIV patients, all were Muslims. On the other hand, the control group consisted of Muslims not willing to do Ramadan fasting and non-Muslims. After 2 weeks of follow-up, 1 patient in the fasting group was dropped out from the study because he had fasted for less than 14 days. Data were analyzed for 29 participants in the fasting group and 29 in the control group. The median age was 35 years old (IQR = 33; 37.25), and the median CD4 cell count was 398 cells/mm^3^ (IQR = 307; 512.50).

At the baseline, age, route of HIV transmission, ART combination, duration of ART, hepatitis B or hepatitis C status, HADS score, antioxidant supplement consumption, CD4 cell count, HIV-RNA, and hs-CRP concentration did not differ significantly between the fasting and control groups (Table 1). At the baseline, the fasting group smoked more cigarettes per day. Body mass index, waist circumference, and baseline TAOS were lower in the fasting group. No participant was receiving treatment for an opportunistic infection.

**TABLE 1:** Baseline characteristics of the fasting and control groups.

Characteristics	Fasting (*n* = 29)	Control (*n* = 29)	*P*-value
Age, years (IQR)	35 (33.5; 38)	35 (32; 37)	0.702
Route of HIV transmission, n (%)			
History of IDU (+)	5 (1.20)	11 (37.90)	0.080
History of IDU (−)	24 (82.80)	18 (62.10)	
ART combination, n (%)			
AZT + 3TC + NVP	12 (41.40)	12 (41.40)	1.000
AZT + 3TC + EFV	9 (31)	9 (31)	
TDF + 3TC + NVP	4 (13.80)	4 (13.80)	
TDF + 3TC/FTC + EFV	4 (13.80)	4 (13.80)	
Duration of ART, n (%)			
<2 years	8 (27.60)	8 (27.60)	1.000
≥2 years	21 (72.40)	21 (72.40)	
Hepatitis B, n (%)	2 (6.90)	4 (13.80)	0.670
Hepatitis C, n (%)	16 (55.20)	17 (58.60)	0.790
Antioxidant supplement consumption, n (%)	7 (24.10)	12 (41.40)	0.160
Number of cigarettes smoked per day	6 (1; 12)	1 (0; 6)	0.041
Body mass index, kg/m^2^ (SD)	21.3 (2.48)	23.43 (3.80)	0.014
Waist circumference, cm (SD)	79.64 (8.39)	85.55 (9.21)	0.013
A-HADS (IQR)	2 (2; 4)	3 (2; 5)	0.135
D-HADS (IQR)	1 (1; 3)	2 (1; 2.50)	0.726
CD4 cell count, cells/μL (IQR)	379 (280.50; 481)	423 (321.5; 519)	0.370
HIV-RNA ≤ 400 copies/ml, n (%)	28 (96.55)	28 (96.55)	1.000
Baseline hs-CRP, mg/L (SD)	0.15 (0.52)	0.12 (0.46)	0.800
Baseline total antioxidant status, mmol/L (IQR)	1.47 (1.34; 1.58)	1.63 (1.48; 1.83)	0.005

Data are given as median [interquartile range (IQR)] or mean [standard deviation (SD)] values. IDU, injecting drug user; ART, antiretroviral therapy; AZT, zidovudine; 3TC, lamivudine; NVP, nevirapine; EFV, efavirenz; TDF, tenofovir; A-HADS, anxiety-hospital anxiety and depression scale; D-HADS, depression-hospital anxiety and depression scale; CD, cluster of differentiation; HIV-RNA, human immunodeficiency virus-ribonucleic acid; hs-CRP, high sensitivity-C reactive protein.

### 3.2. Changes during the follow up period

During the 2-week follow-up, ART adherence did not change in either group. Caloric intake decreased in the fasting group during the 2 weeks of fasting, but this decrease was not significantly different from the non-fasting group (Table 2). Changes in the intake of carbohydrate, protein, lipid, cholesterol, vitamin A, vitamin E, vitamin C, and carotene also did not differ significantly between the fasting and control group after the 2 weeks. Intake of polyunsaturated fatty acid (PUFA) decreased significantly in the fasting group compared to the control group (*p* = 0.012).

**TABLE 2:** Changes in calorie, macronutrient, and antioxidant intake after 2 weeks.

	Fasting (*n* = 29)					Non-fasting (*n* = 29)					*P*	95% CI
	**Pre**	**Post**	***P***	**95% CI**	**Change post-pre**	**Pre**	**Post**	***P***	**95% CI**	**Change post-pre**		
Energy (kcal/day)	1357.28 ± 279.93	1248.97 ± 335.76	0.112	−26.70; 243.31	−108.31 ± 354.94	1220.43 ± 241.88	1249.39 ± 282.44	0.603	−141.55; 83.65	28.95 ± 296.03	0.115	−309.18; 34.67
Carbohydrate (%)	59.50 ± 5.42	62 ± 6.05	0.111	−5.80; 0.62	2.59 ± 8.45	58.13 ± 8.16	59.14 ± 5.66	0.601	−4.93; 2.91	1.01 ± 10.31	0.527	−3.38; 6.53
Protein (%)	14 ± 2.10	13.13 ± 1.96	0.051	−0.006; 1.86	−0.93 ± 2.46	14.79 ± 3.61	15 ± 2.67	0.714	−1.39; 0.96	0.21 ± 3.10	0.125	−2.61; 0.32
Lipid (%)	26.43 ± 5.31	24.06 ± 4.86	0.11	−0.57; 5.30	−2.37 ± 7.73	26.45 ± 7.04	25.75 ± 5.19	0.675	−2.70; 4.10	−0.71 ± 8.98	0.453	−6.06; 2.74
Cholesterol (mg)	262.92 ± 138.86	174.49 ± 102.91	0.001	37.88; 138.98	−88.44 ± 132.89	247.60 ± 149.49	207.72 ± 141.02	0.183	−20.01; 99.78	−39.88 ± 157.46	0.21	−125.19; 28.09
PUFA (g)	11.23 ± 5.52	7.83 ± 3.64	0.012	0.79; 6	−3.40 ± 6.85	9.10 ± 4.15	9.46 ± 4.95	0.743	−2.60; 1.87	0.36 ± 5.89	0.029	−7.12; −0.40
Vitamin A (μg)	483.50 (269; 986.51)	601.90 (262.83; 1180.93)	0.954	0.948; 0.959	−49.63 (−305.37; 377.05)	530.60 (245.65; 667)	461.83 (295.21; 805.68)	0.918	0.911; 0.925	84.84 (−374.71; −332.06)	0.913	0.906; 0.92
Carotene (mg)	0.001 (0.001; 0.40)	0.13 (0.001; 0.53)	0.487	0.474; 0.50	0 (−0.20; 0.40)	0.001 (0.001; 0.166)	0.001 (0.001; 0.166)	0.644	0.631; 0.656	0 (−0.08; 0.17)	0.595	0.582; 0.607
Vitamin E (mg)	4.10 (2.63; 5.15)	2.96 (2.43; 4.08)	0.067	0.06; 0.073	−0.77 (−2.05; 0.57)	3.56 (2.40; 4.43)	3.36 (1.96; 4.61)	0.848	0.839; 0.857	−0.10 (−0.63; 1.42)	0.172	0.162; 0.181
Vitamin C (mg)	29.80 (14.20; 87.23)	40.06 (23.23; 95.70)	0.019	0.016; 0.023	11.83 (−6.95; 32.95)	49.60 (26.98; 153.45)	60.83 (19.51; 204.45)	0.869	0.86; 0.877	8.47 (−30.29; 31.55)	0.222	0.211; 0.233

Data are presented as the mean ± standard deviation or median (interquartile range). PUFA, polyunsaturated fatty acid.

Changes in physical activity, Anxiety-HADS score, and Depression-HADS score did not differ between groups after 2 weeks (Table 3). The number of cigarettes smoked per day and body weight did decrease significantly in the fasting group after 2 weeks of fasting compared to the control group (*p* < 0.001). The total sleep hours per day decreased significantly in the fasting group compared to the control group (*p* = 0.024).

**TABLE 3:** Changes in physical activity, smoking habits, anxiety, depression, and sleep quality after 2 weeks.

	Fasting (*n* = 29)					Non-fasting (*n* = 29)					*P*	95% CI
	**Pre**	**Post**	***P***	**95% CI**	**Change post-pre**	**Pre**	**Post**	***P***	**95% CI**	**Change post-pre**		
Physical activity, METS/day	1.46 (1.40; 1.65)	1.48 (1.36; 1.56)	0.171	0.168; 0.187	0 (−0.12; 0.01)	1.47 (1.38; 1.58)	1.45(1.38; 1.55)	0.271	0.260; 0.283	0 (0; 0.005)	0.093	0.086; 0.101
Number of cigarettes smoked per day	6 (1–12)	3 (0–4)	<0.001	0; 0	−1 (−6; 0)	1 (0; 6)	1 (0; 6)	1	1; 1	0 (0; 0)	<0.001	0; 0
A-HADS	2 (2; 4)	3 (2; 4)	0.383	0.370; 0.395	0 (−1; 1)	3 (2; 5)	3 (2; 4)	0.386	0.374; 0.399	0 (−1; 1)	0.294	0.282; 0.306
D-HADS	1 (1; 3)	1 (1; 3)	0.751	0.739; 0.762	0 (−1; 1)	2 (1; 2.5)	2 (1; 3)	0.884	0.876; 0.892	0 (−1; 0.5)	0.622	0.610; 0.635
PSQI score	3 (2; 5)	4 (3; 6)	0.103	0.950; 0.111	1 (−0.5; 2)	5 (4; 7)	5 (3; 7)	0.661	0.648; 0.673	0 (−1; 0)	0.025	0.021; 0.029
Sleep hours per day	7 (6; 8)	5 (4; 6)	<0.001	0; 0	−2 (−3; −0.5)	6 (5–8)	6 (5–8)	0.491	0.478; 0.504	0 (0; 0)	<0.001	0; 0
Body weight (kg)	61.62 ± 8.19	59.64 ± 8.28	<0.001	1.44; 2.52	−2 (−2.75; −1)	68.42 ± 9.57	68.27 ± 9.96	0.745	−0.77; 1.07	0 (−1.25; 0.65)	0.001	−2.88; −0.78

Data are presented as the mean ± standard deviation or median (interquartile range). METS, metabolic equivalents; A-HADS, anxiety-hospital anxiety and depression scale; D-HADS, depression-hospital anxiety and depression scale; PSQI, Pittsburgh sleep quality index.

### 3.3. Serum hs-CRP concentration and TAOS after the 2-week follow-up

The participants were followed for at least 2 weeks. Final assessment was done in the third or fourth week of Ramadan. Because hs-CRP and TAOS data were not normally distributed, analysis was done using non-parametric test. After the 2-week follow-up (Table 4), hs-CRP concentration decreased significantly (*p* = 0.011) in the fasting group (–0.41 mg/L [IQR = –1 to 0.10]) compared to the control group (0.20 mg/L [IQR = –0.30; 1.50]). TAOS increased in both groups even though the changes were not significant (0.05 mmol/L [IQR = –0.03; 0.12] vs. 0.04 mmol/L [IQR = –0.13; 0.36], fasting vs. non-fasting, *p* = 0.408). The post-Ramadan fasting hs-CRP variable was then log-transformed to be entered in a linear regression model predicting the change of hs-CRP after Ramadan fasting. Ramadan fasting did explain a significant amount of the variance in hs-CRP [*F*_(1, 56)_ = 6.26, *p* = 0.015, *R*^2^ = 0.101]. The regression coefficient (B = −0.317, 95% CI −0.636; −0.07) indicated that Ramadan fasting decrease hs-CRP value by 0.317 points.

**TABLE 4:** Changes in main outcomes (hs-CRP and total antioxidant status) after 2 weeks.

	Fasting (*n* = 29)					Non-fasting (*n* = 29)					*P*	95% CI	*R* ^2^	B (95% CI)
	**Pre**	**Post**	***P***	**95% CI**	**Change post-pre**	**Pre**	**Post**	***P***	**95% CI**	**Change post-pre**				
hs-CRP (mg/L)	1.30 (0.60; 2.65)	0.80 (0.30; 1.60)	0.011*	0.008; 0.014	−0.41 (−1; 0.10)	1.20 (0.6; 3.5)	1.90 (0.6; 4.8)	0.097	0.910; 0.106	0.20 (−0.30; 1.50)	0.004*	0.002; 0.006	0.101	−0.317 (−0.636; −0.070)*
Total antioxidant status (mmol/L)	1.47 (1.34; 1.58)	1.50 (1.40; 1.59)	0.089	0.081; 0.096	0.05 (−0.03; 0.12)	1.63 (1.47; 1.83)	1.80 (1.62; 1.89)	0.21	0.209; 0.23	0.04 (−0.13; 0.36)	0.408	0.396; 0.421	–	–

*p < 0.05. Data are presented as the mean ± standard deviation or median (interquartile range). hs-CRP, high sensitivity-C reactive protein.

## 4. Discussion

To our knowledge, this is the first study on the effects of Ramadan fasting on hs-CRP levels and TAOS in HIV patients on ART. In our study, Ramadan fasting reduced some indicators of inflammation in HIV patients on ART. At the baseline, hs-CRP concentration did not differ between the fasting and control groups, but it did decrease significantly in the fasting group after the 2 weeks of Ramadan fasting.

Our data are consistent with the results of other studies on hs-CRP levels and fasting, such as those by Shariatpanahi et al. (4) on patients with metabolic syndrome and by Askari et al. (7) on asthmatic patients. By contrast, our results differ from those of Fawzi et al. (8), who reported increased hs-CRP levels during Ramadan fasting in schizophrenic patients. This difference may be explained by the increases in caloric intake and body weight observed during Ramadan fasting in their study. Their study was also limited by the absence of a control group and physical activity record.

HIV infection is associated with chronic immune activation and pro-inflammatory conditions (9). Studies have shown that inflammatory parameters in HIV patients are higher than in the general population. Antiretroviral therapy does not entirely improve this pro-inflammatory condition (1, 10). Inflammation is associated with endothelial dysfunction in HIV patients who received ART and patients who have not (11).

People living with HIV also have higher levels of monocyte activation and altered coagulation than people who do not have HIV. Many of these biomarkers are linked to atherogenesis, an inflammatory process that leads to atherosclerotic cardiovascular disease (12, 13).

Studies have found that people living with HIV have a higher risk of cardiovascular disease than people who do not have HIV. HIV-specific risk factors and classic and non-traditional cardiovascular disease risk factors are among the mechanisms driving this risk (13). Among the established risks are elevated hs-CRP values. Elevated hs-CRP in HIV patients is associated with non-AIDS events [cardiovascular disease, non-AIDS-related malignancies, diabetes, and lung disease (11, 14)]. The significant correlation between hsCRP and cardiovascular disease is likely due to its strong correlation with other established risk factors such as smoking, diabetes, visceral obesity, and inflammation markers (15). Moreover, high hs-CRP is independently associated with atherosclerotic cardiovascular disease including in patients with good atherogenic lipid level (16). Therefore, high inflammation markers should be one of the concerns in the HIV care continuum. The decrease in hs-CRP levels due to the influence of Ramadan fasting is expected to reduce the risk of non-AIDS events, especially cardiovascular disease in HIV patients.

The significant reduction of hs-CRP concentration in the fasting group observed in our study was not related to total caloric intake, macronutrient intake, micronutrient intake (i.e., vitamin A, vitamin E, vitamin C, and carotene intake), physical activity, or anxiety or depression level during Ramadan fasting because no significant changes in these parameters were observed during fasting. Although caloric intake and physical activity did not change in the fasting group, body weight decreased after the 2 weeks of Ramadan fasting.

Ramadan fasting is a unique intermittent fast. During Ramadan fasting, people are prohibited from eating, drinking, and smoking from dawn until sunset. Although the total caloric intake per day may not change, the frequency of large meal portions decreases from 3 times to twice daily and the timing of meals also changes. These changes can affect metabolism and the circadian system and can attenuate inflammation, as shown by previous studies (17, 18). A study by Kahleova et al. (17) of people with type 2 diabetes mellitus showed that a diet of 2 large-portion meals resulted in greater reduction in body weight and improvement in insulin sensitivity than 6 small-portion meals did. Intermittent fasting can modify brain neurochemistry and neural activity to optimize brain function and peripheral energy metabolism. Another study showed that intermittent fasting increased leptin and insulin sensitivity, reduced leptin and insulin levels, reduced insulin-like growth factor 1, and increased adiponectin and ghrelin, which are factors that can attenuate inflammation (18).

In our study, PUFA intake decreased significantly during fasting. PUFA intake included omega-3 PUFA (α-linolenic acid) and omega-6 PUFA (linoleic acid). An increased ratio of omega-6 to omega-3 is related to increased cardiovascular risk (19). A previous study by Hanafiah et al. (20) showed that the ratio of omega-6 to omega-3 consumption is high in Indonesians: 46.1 ± 6.1 in coronary heart disease populations and 31.2 ± 2.1 in the healthy population. Decreased PUFA intake in the fasting group in our study may reflect a decrease in omega-6 intake. The lower intake of omega-6, which is proinflammatory, may have contributed to the observed decrease in hs-CRP concentration.

The significant decrease in the number of cigarettes smoked per day may also have contributed to the reduction in hs-CRP concentration in the fasting group. A study by Jamal et al. (21) showed that serum hs-CRP levels are higher in smokers than in non-smokers.

In our study, the number of total sleep hours per day decreased in the fasting group. A previous study by Meier-Ewert et al. (22) showed that shorter total sleep duration is related to higher hs-CRP levels. The contrasting finding in our study might reflect the influence of other factors on hs-CRP levels.

Although TAOS increased more in the fasting group than in the control group, the difference between groups was not significant. Future studies with longer observation periods and larger samples are needed to confirm the changes in TAOS.

Our study has several strengths, such as including a control group whose members did not fast and recording caloric intake, physical activity, and smoking habits. However, our study does have some limitations. The improvement in inflammatory markers in the fasting group may be attributed to changes in the frequency and timing of large-portion meals, PUFA intake, total cigarette smoking per day, or body weight. We could not determine which of the observed changes led to the decreased hs-CRP concentration because of the small sample size.

Additionally, blood sampling is only done before fasting and during Ramadan fasting. It is advisable to take a third sample after Ramadan fasting to see if the hs-CRP level increases or the TAOS level decreases again outside Ramadan. Another limitation of our study was that it did not exclude patients taking antioxidant supplements and still smoking. This is a factor that can affect the TAOS results obtained.

In conclusion, we found that hs-CRP concentration was lower after 2 weeks of Ramadan fasting in HIV patients on ART. This result suggests that Ramadan fasting may reduce inflammation caused by chronic immune response activation in HIV patients on ART.

## Data availability statement

The raw data supporting the conclusions of this article will be made available by the authors, without undue reservation.

## Ethics statement

The studies involving human participants were reviewed and approved by Research Ethics Board of the Faculty of Medicine, University of Indonesia and the Research Ethics Board of Dr. Cipto Mangunkusumo Hospital, Jakarta, Indonesia. The patients/participants provided their written informed consent to participate in this study.

## Author contributions

AW, EY, SS, and FW: research design, manuscript drafting, data analysis, and interpretation. AW and TK: data collection. All authors manuscript revision, read, and approved the final version of the manuscript.
